# Postmortem Redistribution of Drugs Commonly Used in Rapid Sequence Induction for Anesthesia: A Review

**DOI:** 10.3390/jcm15041622

**Published:** 2026-02-20

**Authors:** Sofia Gkarmiri, Sofia-Chrysovalantou Zagalioti, Efstratios Karagiannidis, Panagiotis Zagaliotis, Panagiotis Stachteas, Aikaterini Apostolopoulou, Sotirios Charalampos Diamantoudis, Marios G. Bantidos, Christos Kofos, Katerina Kotzampassi, Vasileios Grosomanidis, Nikolaos Raikos, Barbara Fyntanidou

**Affiliations:** 1 Department of Emergency Medicine, AHEPA University General Hospital, Aristotle University of Thessaloniki, 541 24 Thessaloniki, Greece; sofia.gkarmiri@gmail.com (S.G.); sofia_zag@yahoo.com (S.-C.Z.); aapostoo@auth.gr (A.A.); mbadidos@gmail.com (M.G.B.); chriskofos21@gmail.com (C.K.); bfyntan@yahoo.com (B.F.); 2 Transplantation/Oncology Program, Division of Infectious Diseases, Weill Cornell Medicine, New York, NY 10065, USA; paz4002@med.cornell.edu; 3 Second Department of Cardiology, Hippokration General Hospital, Aristotle University of Thessaloniki, 541 24 Thessaloniki, Greece; staxteasp@gmail.com; 4 School of Pharmacy, Faculty of Health Sciences, Aristotle University of Thessaloniki, 541 24 Thessaloniki, Greece; s.c.diamantoudis@gmail.com; 5 Theageneio Anticancer Hospital of Thessaloniki, 546 39 Thessaloniki, Greece; 6 Department of Surgery, Aristotle University of Thessaloniki, 541 24 Thessaloniki, Greece; kakothe@yahoo.com; 7 Department of Anesthesiology and ICU, Faculty of Medicine, Aristotle University of Thessaloniki, 541 24 Thessaloniki, Greece; grosoman@otenet.gr; 8 Laboratory of Forensic Medicine and Toxicology, Medical School, Aristotle University of Thessaloniki, 541 24 Thessaloniki, Greece; raikos@auth.gr

**Keywords:** postmortem redistribution, rapid sequence induction, sedatives, intravenous anesthetics, neuromuscular blockade agents, midazolam, fentanyl

## Abstract

**Background:** Rapid Sequence Induction (RSI) is a widely used method for emergency airway management in critically ill and clinically unstable patients. Beyond the risks inherent to the procedure itself, RSI is almost exclusively performed in emergency settings where patients present with severe physiological derangement and a high risk of aspiration. In postmortem examinations, forensic toxicology results may be influenced by the patient’s clinical condition, the sampling site, the postmortem interval (PMI), and postmortem drug redistribution (PMR). This review aims to evaluate the existing literature regarding PMR of drugs commonly used during RSI. **Methods:** PubMed/MEDLINE, Embase and the Cochrane Library were searched for studies on PMR of drugs used in intravenous (IV) RSI (up to November 2025). Human and animal studies, patient populations comparable to critically ill individuals requiring RSI, and forensic case reports of exclusively IV drug administration were included. Studies on recreational use, overdose and non-IV administration were excluded. **Results:** Data on the PMR of IV-administered RSI drugs remain limited. Most available studies involve Intensive Care Unit (ICU) patients or individuals who underwent RSI in emergency settings. Fentanyl and midazolam appear to demonstrate notable PMR. Several factors influencing postmortem drug concentrations were identified. Although these findings are consistent with the existing literature, the small number of studies and the heterogeneity of data preclude definitive conclusions. **Conclusions:** Critical patient condition, including frailty due to advanced age, hemodynamic instability (particularly in ICU patients), hypoalbuminemia, body mass index (BMI), and injury and/or trauma, as well as the interval between IV drug administration and death, appear to affect postmortem concentrations of drugs used during RSI. The potential for PMR of certain agents, such as fentanyl and midazolam, adds further complexity. Given the scarcity of consolidated evidence and until further research provides more robust data, postmortem drug levels should not be interpreted as directly reflective of antemortem concentrations.

## 1. Introduction

The emergency management of critically ill patients, particularly those with hemodynamic instability, is inherently challenging and requires immediate, coordinated interventions to stabilize the patient and prevent cardiopulmonary arrest [1]. Among the initial interventions, emergency airway management is a foremost priority. Rapid Sequence Induction (RSI)—also referred to as Rapid Sequence Intubation or Rapid Sequence Induction and Intubation (RSII)—constitutes a standardized technique for securing the airway rapidly while minimizing the risk of regurgitation and pulmonary aspiration [2]. Its use is particularly relevant in patients with either inadequate or unknown fasting status or underlying conditions that predispose them to aspiration, irrespective of fasting [3,4]. It is achieved through the sequential administration of potent, fast-acting anesthetic agents and neuromuscular blockers, thereby shortening the critical interval between loss of protective airway reflexes and endotracheal intubation [2].

Typically, RSI involves the administration of an intravenous (IV) anesthetic or sedative in combination with a neuromuscular blocking agent, with the optional addition of an opioid to optimize hemodynamic stability and analgesia [5]. The choice of agents and their dosages during RSI should be individualized according to the patient’s clinical condition and hemodynamic status [5]. In current practice, commonly employed IV anesthetics in RSI include etomidate, ketamine, propofol and midazolam, while thiopental, historically used, has largely been phased out due to prolonged recovery time and adverse cardiovascular effects [6,7,8,9]. Commonly used neuromuscular blockers comprise rocuronium, vecuronium and suxamethonium (succinylcholine) [6,7,8]. Opioid adjuncts, most notably fentanyl, may be administered to attenuate sympathetic responses during induction [10,11].

Patients undergoing RSI in emergency and critical care settings often present with substantial baseline mortality risk due to underlying critical illness, hemodynamic instability, major trauma, or other life-threatening conditions rather than the procedure itself [12]. Consequently, adverse outcomes, including peri-intubation cardiac arrest, must be interpreted within this complex clinical environment, where the patient’s unknown medical history, physiological derangements and multiple confounding factors coexist with the urgency and inherent risks of interventions such as RSI [13,14].

Given that many patients undergoing RSI ultimately succumb to their underlying critical illness, either during resuscitation or shortly thereafter, the subsequent forensic interpretation of toxicological findings becomes particularly important—especially when anesthetic and neuromuscular blocking agents have been administered in the perimortem period [1]. Although forensic toxicology has noted significant technical advancement over recent years, the interpretation of postmortem toxicological findings continues to pose considerable challenges [15]. Knowledge of the patient’s individual history—including age, general health status and drug use history—is essential because these variables may substantially influence antemortem pharmacokinetics [16]. Establishing a reliable correlation between postmortem and antemortem drug concentrations is difficult, given the scarcity of human data and the limited applicability of animal studies [17]. Other important considerations include the site of blood sampling and the time interval between death and specimen collection (postmortem interval (PMI)), both of which can significantly affect measured concentrations [18].

Postmortem drug redistribution (PMR) is a well-described phenomenon in forensic toxicology, referring to site- and time-dependent concentration changes after death [19,20]. These changes may occur through diffusion of drugs from tissues with high concentrations to surrounding compartments with lower concentrations, thereby complicating the interpretation of measured postmortem levels [19,20]. Elevated postmortem levels may be mistakenly regarded as reflecting fatal antemortem exposure, potentially leading to an erroneous attribution of intoxication as the cause of death [21]. The mechanisms underlying PMR include redistribution from drug reservoirs such as highly perfused organs and adipose tissues, cadaveric biomechanical changes (cell death, membrane breakdown), blood coagulation, postmortem blood heterogeneity, and microbial enzymatic activity, as well as physicochemical and pharmacokinetic properties of each drug—such as pKa, lipophilicity, protein binding, volume of distribution, and metabolic pathways [18].

In the context of RSI, additional factors complicate toxicological interpretation. Hemodynamic instability and critical illness can substantially affect drug distribution, metabolism and clearance [16]. Moreover, the patient’s drug use history is often unknown, and some RSI agents—such as fentanyl, propofol and ketamine—are also used recreationally, introducing the possibility of pre-existing drug exposure [22]. Concurrent administration of multiple agents during RSI may further alter toxicological profiles through pharmacodynamic synergy, pharmacokinetic interactions or altered absorption [23].

Despite their frequent clinical use, consolidated data on the postmortem behavior of RSI drugs remain limited. Significant knowledge gaps persist regarding postmortem redistribution patterns of RSI agents, their tissue distribution and their impact on forensic interpretation. This review aims to synthesize current evidence on factors influencing PMR of drugs commonly used in RSI, examine known patterns of postmortem tissue distribution and discuss the practical implications for forensic toxicology.

## 2. Literature Search Strategy

This narrative review was guided by a comprehensive and structured literature search aimed at identifying data on the PMR of drugs commonly used in RSI. Searches were conducted in PubMed/MEDLINE, Embase and the Cochrane Library from database inception. Two authors (S.G. and S.-C.Z.) independently conducted the literature search up to 5 November 2025. Disagreements were resolved by discussion, or by consultation with a third author (E.K.) if consensus could not be reached. The search strategy combined controlled vocabulary terms (MeSH). This set of terms comprised “postmortem redistribution”, “postmortem distribution”, “postmortem pharmacokinetics”, “sedative drugs”, “neuromuscular blockade”, “rapid sequence induction”, “propofol”, “ketamine”, “midazolam”, “fentanyl”, “etomidate”, “rocuronium”, “vecuronium”, “succinylcholine”, “suxamethonium”, “thiopental” and “thiopentone”. The full PubMed search strategy is provided in Table 1.

**Figure 1:**
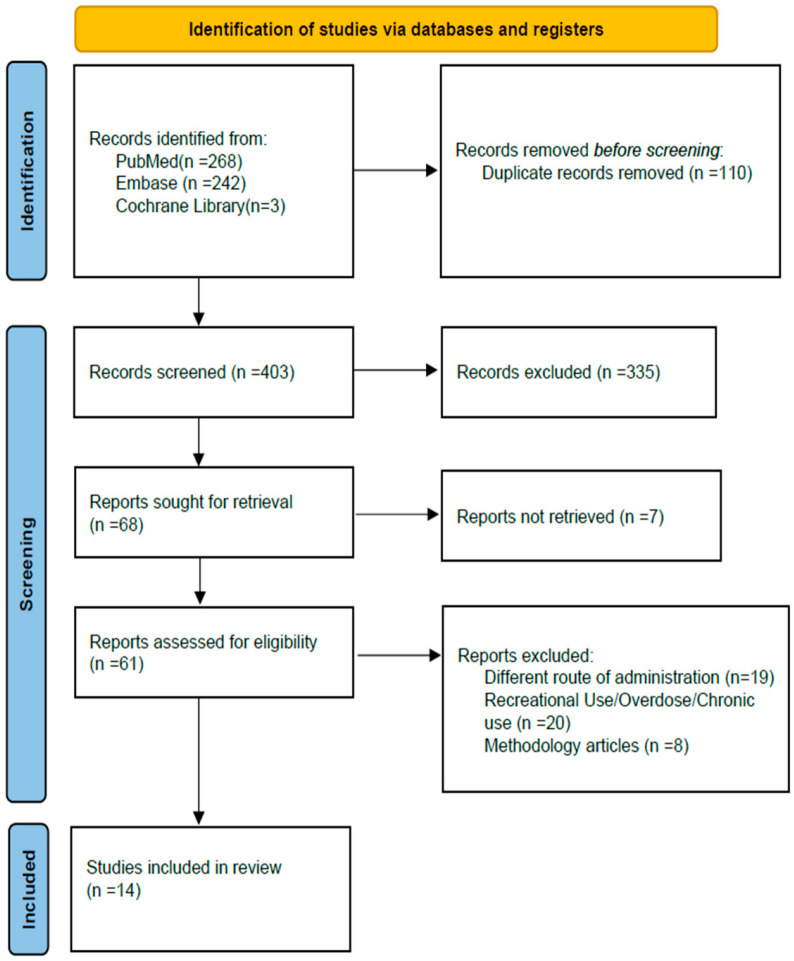
PRISMA flow diagram of the study.

Inclusion criteria were human or animal studies reporting PMR following IV administration of drugs used for RSI; studies involving patient populations comparable to critically ill individuals requiring RSI, including ICU sedation studies in critically ill patients where continuous IV infusions were used as proxies for IV bolus exposure; and forensic case reports involving drugs administered exclusively IV, independent of the clinical context or indication for use. Exclusion criteria were studies focusing on recreational drug use, overdose and chronic transdermal therapy. A PRISMA flow diagram summarizing the study selection process is shown in Figure 1.

## 3. Pharmacological Categories

### 3.1. Intravenous Anesthetics

#### 3.1.1. Midazolam

*Patient Population—Route of Administration*

Midazolam has been studied in critically ill patients and emergency care settings . In an Intensive Care Unit (ICU) cohort, the drug was administered as a continuous IV infusion [24]. In an emergency or urgent procedural cohort, midazolam was also administered IV, although dosing information was not documented [25]. Another similar study with documented administration times and doses showed that a higher body mass index (BMI) was associated with higher central-to-peripheral (C/P) ratios (*p* = 0.027) [26]. In the study, individuals with a low BMI were few, resulting in a limited distribution across BMI categories. In a different ICU cohort characterized by severe hemodynamic instability, midazolam was administered IV and its concentrations varied across cardiac blood, pericardial fluid and bone marrow aspirate [27].

*Concentrations*

In a prospective single-center ICU study, antemortem, perimortem (within 1 h of death), and postmortem blood samples were analyzed [24]. Perimortem midazolam concentrations were higher than antemortem levels (median time from administration to death: 3 h). Postmortem midazolam concentrations generally decreased compared with perimortem values (*p* = 0.07).

*Sampling Site*

Perimortem samples were collected from peripheral veins, arteries and central lines, while postmortem samples were collected from the aortic arch, femoral vein and jugular vein [24]. Stratification by sampling site was not performed due to small group sizes. Additional data indicated that midazolam concentrations differed depending on sampling site and the interval from IV administration to death: heart concentrations were highest with incomplete distribution, whereas brain, liver or kidney concentrations were higher with longer intervals [25]. Pericardial fluid and bone marrow aspirate were also evaluated as alternative postmortem matrices; midazolam concentrations were generally lower in pericardial fluid (*p* < 0.0001) than in bone marrow or cardiac blood [27].

*PMI*

Comparison of mortuary admission and autopsy blood samples showed an increase in midazolam concentrations of less than 10%, which was not statistically significant (*p* ≥ 0.05); the number of cases analyzed for midazolam was small [28].

#### 3.1.2. Thiopental

*Patient Population—Route of Administration*

Thiopental has been studied in critically ill ICU patients. In a single-center cohort study, most patients were severely ill, with a notable proportion having pre-existing liver or kidney dysfunction. The drug was administered as a continuous IV infusion [24].

*Concentrations*

Postmortem trends for thiopental are inconclusive. In an ICU cohort, no systematic postmortem redistribution was observed [24]. In a procedural sedation case, thiopental concentrations were higher in brain and thymus compared with peripheral tissues, but overall concentrations remained low [29].

*Sampling Site*

Postmortem thiopental concentrations were also measured at multiple blood sites, but trends were not conclusive [24].

*PMI*

No PMI data were reported for thiopental in the reviewed studies.

#### 3.1.3. Etomidate

*Patient Population—Route of Administration*

These data were not reported in the reviewed studies.

*Concentrations*

In a case of fatal intoxication, postmortem femoral blood concentration was 0.40 mg/L, slightly above the therapeutic range (0.082–0.32 mg/L) [30]. In cases where etomidate was administered therapeutically (for anesthesia and other medical interventions) two hours prior to death, femoral blood concentrations were markedly lower (<0.026–0.05 mg/L) [30].

*Sampling Site*

Data on sampling site were not reported in the reviewed studies.

*PMI*

Data on PMI were not reported in the reviewed studies.

#### 3.1.4. Propofol

*Patient Population—Route of Administration*

Propofol has been studied in critically ill patients. In a single-center cohort, the drug was administered as a continuous IV infusion [24], and in a separate critically ill population with marked hemodynamic instability, postmortem concentrations varied across cardiac blood, pericardial fluid and bone marrow aspirate [27].

*Concentrations*

Postmortem concentration trends for propofol were not conclusive [24].

*Sampling Site*

Postmortem propofol concentrations were lower in pericardial fluid compared with bone marrow aspirate and cardiac blood (*p* < 0.001), although available data remain limited [27].

*PMI*

Propofol was detected at 0.37 μg/mL seven days after a 19 h continuous IV infusion in a case report, reflecting tissue distribution and delayed release, without relation to the cause of death [31].

#### 3.1.5. Ketamine

*Patient Population—Route of Administration*

Ketamine has been studied in critically ill ICU patients and an in vivo study using rats [24,32].

*Concentrations*

Postmortem concentration trends for ketamine were not conclusive [24].

*Sampling Site*

In an in vivo study, ketamine (40 mg/kg IV) and its metabolite norketamine were measured in vitreous and aqueous humor as well as ocular tissues perimortem and 17 h postmortem [32]. Postmortem blood ketamine levels decreased, while concentrations in ocular tissues—particularly retina and choroid—increased (*p*-value ranged from <0.001 to <0.05 in the different ocular tissues); norketamine showed a similar trend (*p* < 0.05).

*PMI*

Data on PMI were not reported in the reviewed studies.

### 3.2. Neuromuscular Blocking Agents

#### Suxamethonium (Succinylcholine)

Data on succinylcholine are derived from a single controlled in vivo study on guinea pigs [33].

*Patient Population—Route of Administration*

Guinea pigs were administered 40 mg/kg succinylcholine IV.

*Concentrations*

Low postmortem concentrations were found in muscle (5 pmol/g), the kidney (5–1500 pmol/g) and urine (5–650 pmol/mL), whereas ocular tissues showed the highest concentrations (280 + 36 pmol/g), which decreased after six days and became undetectable four weeks postmortem.

*Sampling Site*

Muscle, the kidney, urine and ocular tissues were analyzed, with ocular tissues retaining detectable levels for the longest period.

*PMI*

PMI influenced succinylcholine detection, with concentrations declining over time in ocular tissues.

### 3.3. IV Opioids Used in RSI

#### Fentanyl

*Patient Population—Route of Administration*

Studies have examined fentanyl in critically ill ICU and hospitalized patients. One ICU cohort study analyzed critically ill patients, with a substantial proportion having pre-existing liver or kidney failure, and most died from circulatory or multi-organ failure [24]. Fentanyl was administered via continuous IV infusion. Other studies included patients receiving fentanyl IV or transdermally, with no statistically significant difference between the two routes (*p* = 0.134) [34,35], with brain-to-blood ratios showing minor differences between administration routes [35]. Single-case and case series reports describe patients receiving IV fentanyl during hospitalization, showing minimal differences between antemortem and postmortem concentrations [19,36].

*Concentrations*

Postmortem fentanyl concentrations exhibit variable redistribution depending on the clinical context and patient condition [37,38]. In ICU patients, a recent prospective study of 46 individuals reported notable postmortem increases (*p* = 0.002), reflecting both therapeutic administration and critical illness, sometimes sufficient to influence toxicological interpretation [24]. In contrast, minimal postmortem increases were reported in a single-case study with a C/P ratio close to unity [36].

*Sampling Site*

Postmortem fentanyl concentrations are influenced by the site of sample collection. In a study by Lennborn et al. of ICU patients, perimortem samples were obtained from peripheral veins, arteries or central venous lines, whereas postmortem samples were collected from the aortic arch, femoral vein and jugular vein [24]. Tissue samples such as liver and kidney often demonstrate higher fentanyl concentrations than peripheral blood, supporting the use of liver-to-peripheral blood (L/P) ratios as a marker of postmortem redistribution, potentially more reliable than the C/P ratio [34,39]. Brain tissue has also been proposed as an alternative sampling site and appeared less affected by redistribution, regardless of suspected intoxication [35].

*PMI*

PMI has been examined in several studies assessing fentanyl stability and its potential for time-dependent redistribution. In an ICU cohort study, no meaningful association was identified between PMI duration and postmortem changes [24]. In contrast, Brockbals et al. demonstrated that PMI can contribute to significant temporal increases in fentanyl concentrations when consecutive postmortem samples are obtained [34], highlighting that under certain circumstances, fentanyl may undergo measurable redistribution over time. Case reports and small series generally showed minimal PMI-related changes, suggesting limited impact on toxicological interpretation [34,36].

A summary of all studies included in the review and their respective results is provided in Table 2.

## 4. Discussion

This narrative review identified only limited evidence on PMR following IV administration of drugs used for RSI. No studies were available for rocuronium or vecuronium, and only one animal study addressed succinylcholine. The lack of data on neuromuscular blocking agents likely reflects their clinical pharmacology: IV administration induces rapid paralysis of the respiratory and skeletal muscles, preventing adequate systemic distribution and requiring prompt intubation and mechanical ventilation; failure to secure the airway results in circulatory collapse and incomplete antemortem distribution, making PMR evaluation difficult [40,41]. Forensic analysis can identify these agents, but their contribution to death is most probably indirect, due to the inability to ventilate the patient [40,41].

Two animal studies involving IV ketamine and succinylcholine were included to provide partial physiological comparability to humans, though their applicability is limited, as the models used were guinea pigs and rats rather than porcine species, which generally exhibit close physiological similarity to humans [42,43,44]. Several human studies were cited repeatedly because they provide uniquely comprehensive datasets, including patient populations comparable to critically ill individuals requiring RSI, examination of multiple RSI agents, and the rare availability of both antemortem and postmortem samples. Given the scarcity of directly relevant research, these datasets provide disproportionate methodological value. Some included studies involved ICU patients receiving continuous IV sedation. These patients were critically ill and shared key pathophysiological features with those undergoing RSI. Continuous infusions were used as proxies for IV bolus exposure, so the findings should be interpreted with caution.

Fentanyl data were relatively limited despite the extensive literature on its recreational use, overdose and chronic transdermal therapy—areas excluded from this review. Postmortem concentrations after transdermal, oral or intranasal exposure are not comparable to IV administration due to differences in absorption, redistribution, chronic use effects, tolerance, environmental factors and patient characteristics such as hypoalbuminemia and advanced age [36,37,45,46,47]. Overdose or chronic misuse may further elevate postmortem fentanyl via altered pharmacokinetics [36,37]. These differences render such studies unsuitable for an RSI-focused PMR analysis.

Interpretation of postmortem toxicology in patients undergoing RSI is complicated by the interplay of critical illness, perimortem drug administration and PMR. Most reviewed studies involved critically ill patients, mainly in ICUs, approximating the population requiring RSI in emergency settings. These patients frequently exhibited organ dysfunction or circulatory compromise, which can substantially alter drug absorption, distribution, metabolism, and excretion (ADME) [24,46,47]. Those factors that could have potentially led to postmortem concentrations mimicking PMR due to incomplete antemortem distribution (especially for midazolam and fentanyl) in the reviewed studies are summarized in Table 3. Continuous IV infusion, polypharmacy, extremes of BMI, trauma and hypoalbuminemia further complicate the interpretation of postmortem drug concentrations, particularly for lipophilic and protein-bound drugs such as fentanyl and midazolam [26,46,47,48,49,50].

Particularly regarding basic, lipophilic drugs with a volume of distribution (Vd) higher than 3 L/kg, PMR is generally expected; however, this association is not consistently supported across the literature [18,19,33,45,47]. Table 4 demonstrates the core pharmacokinetic determinants and the corresponding possible PMR potential for each drug studied.

Regarding PMR potential, fentanyl evidence remains limited, due to small sample sizes [34] or heterogeneous study populations [24]. Only one case report by Mclntyre et al. reported a C/P ratio slightly above unity [36], consistent with the prior literature suggesting that fentanyl may undergo PMR [37,45]. However, the scarcity of reliable clinical data prevents definitive conclusions. Benzodiazepines generally show minimal PMR, with midazolam as a potential exception, though limited by incomplete documentation of administration-to-death intervals [26]. No L/P ratio data were reported for fentanyl or midazolam, despite evidence that L/P may be more reliable than C/P for predicting PMR [57]. In the controlled in vivo study on guinea pigs [33], succinylcholine was rapidly metabolized in the blood by pseudocholinesterase (butyrylcholinesterase, BChE), resulting in very low plasma concentrations after administration [58]. Ocular tissues, however, contain very low concentrations of this enzyme, allowing the drug to persist longer [58]. This likely explains the higher postmortem concentrations observed in ocular tissue. For other RSI agents, either no PMR data exist or evidence is inconclusive.

PMI effects were inconsistent: some studies found minimal concentration changes [24,28], whereas others reported time-dependent increases, particularly for fentanyl in early postmortem samples [34]. These discrepancies largely reflect methodological heterogeneity, including irregular sampling intervals, small case numbers and critically ill populations with altered pharmacokinetics. For most RSI agents (thiopental, ketamine, propofol), PMI-specific data were absent or inconclusive. Overall, current evidence remains insufficient to define clear PMI-related redistribution patterns after IV RSI drug administration.

In summary, the available evidence suggests that several factors may influence postmortem concentrations of the examined drugs, creating significant potential for misinterpretation as PMR. The reviewed studies indicate that key determinants predominantly relate to antemortem alterations in ADME processes, including critical illness [24,34,46,47], and organ or circulatory failure [46] and factors related to drug administration, including the interval between final administration and death [24,37]. Additional modifiers include polypharmacy and hypoalbuminemia [24], as well as patient-specific characteristics such as BMI [46] and injury or trauma [48]. Other factors supported in the broader literature—such as chronic use or long-term use, substance misuse, overdose, advanced age and variation in the route or method of administration—could also substantially alter postmortem concentrations [37,45,48]. However, these factors could not be meaningfully assessed in the present review due to the limited data availability and the characteristics of the included patient populations. Collectively, these considerations underscore the complexity of interpreting postmortem toxicology in the context of RSI and highlight the need for more robust, targeted research.

*Limitations*

This narrative review is limited by the small number of studies on PMR following IV RSI drugs, most of which were case reports or observational studies. No data were available for rocuronium. Thiopental in RSI has mostly been replaced by other induction agents, but it remains a relatively economical option, particularly in centers with limited resources [59]. Moreover, the wide variability of the degree of correlation between the physicochemical and pharmacokinetic properties of RSI drugs, including lipophilicity, volume of distribution, protein binding and pKa, and the likelihood of PMR further complicates the interpretation of postmortem concentrations and highlights the need for future systematic quantitative synthesis to provide robust and comparable findings. Clinical studies often had small, heterogeneous samples and incomplete antemortem dosing information, reflecting the practical and ethical challenges of conducting controlled research in critically ill patients requiring RSI. Two animal studies were included, but their applicability to humans is limited. Finally, ICU populations differ from emergency RSI patients in drug exposure and physiology. ICU patients were critically ill, similar to those undergoing RSI, sharing key pathophysiological features. However, continuous infusions do not fully mimic the pharmacokinetics of rapid IV bolus administration, so the results should be interpreted with caution.

*Future Directions*

The paucity of data highlights the need for further studies. Future research should focus on comparing antemortem and consecutive postmortem samples, particularly within the first hours after death, and stratifying patients by the interval between drug administration and death. Rigorous planning, interprofessional coordination and detailed documentation will be essential. Controlled animal models, especially porcine, could provide mechanistic insights into drug distribution and PMR, including during critical scenarios such as cardiopulmonary resuscitation.

## 5. Conclusions

This narrative review summarizes all available evidence regarding PMR of drugs commonly used in RSI and the influence of reported factors. Patient-specific factors, particularly critical illness and the interval between IV drug administration and death, appear to affect antemortem distribution and postmortem concentrations. Midazolam and fentanyl show potential for PMR, though available data are scarce and inconsistent. For other RSI agents, no reliable conclusions can be drawn. Until further controlled studies—incorporating consecutive postmortem sampling, rigorous documentation and potentially animal models—are conducted, postmortem drug concentrations should not be interpreted as reflective of antemortem levels.

## Figures and Tables

**Table 1:** PubMed search strategy.

Search Entry—PubMed		
Search	Entry Terms	Result
#1	“postmortem redistribution” OR “postmortem distribution” OR “postmortem pharmacokinetics”	12,234
#2	“sedative drugs” OR “neuromuscular blockade” OR “rapid sequence induction” OR “propofol” OR “ketamine” OR “midazolam” OR “fentanyl” OR “etomidate” OR “rocuronium” OR “vecuronium” OR “succinylcholine” OR “suxamethonium” OR “thiopental” OR “thiopentone”	226,261
#3	#1 AND #2	268

**Table 2:** Summary of all studies included in the review ^1^.

Drugs	Study	Type of Study	Route	Study Population	Results
Anesthetics					
**Midazolam**	Lennborn et al. [24]	Observational	IV	ICU patients	Increased perimortem concentration due to incomplete antemortem distribution
	Oertel et al. [25]	Observational	IV	Patients that underwent emergency/urgent procedures	Time elapsed between administration and death determines the distribution of the drug between sampling sites (cardiac blood, peripheral blood, brain, liver, kidneys), with cardiac blood and brain concentrations being the highest when distribution is deemed incomplete
	Tominaga et al. [27]	Observational	IV	Forensic cases	Postmortem concentrations in the pericardial fluid were significantly lower than bone marrow aspirate and cardiac blood concentrations Insufficient data
	de Groot et al. [26]	Observational	IV	Forensic cases	Higher values of C/P ratio were noted for higher BMI Small number of cases with low BMI
	Gerostamoulos et al. [28]	Observational	IV	Forensic cases	No statistically significant changes Small sample
**Thiopental**	Lennborn et al. [24]	Observational	IV	ICU patients	Inconclusive
	Yasuda et al. [29]	Case report	IV	Patient that underwent procedural sedation	Very low postmortem concentrations in all samples, except for the brain and thymus
**Etomidate**	Molina et al. [30]	Case report	IV	Individual—fatal intoxication	Postmortem concentration just above therapeutic levels in a case of fatal intoxication
**Propofol**	Lennborn et al. [24]	Observational	IV	ICU patients	Inconclusive
	Tominaga et al. [27]	Observational	IV	Forensic cases	Postmortem concentrations in the pericardial fluid were significantly lower than bone marrow aspirate and cardiac blood concentrations Insufficient data
	George et al. [31]	Case report	IV	Patient that underwent a surgical procedure	Propofol detected postmortem 7 days after continuous IV administration
**Ketamine**	Lennborn et al. [24]	Observational	IV	ICU patients	Inconclusive
	Arora et al. [32]	In vivo animal study	IV	Rats	Decrease in postmortem blood concentrations Increased levels of ketamine and norketamine in ocular tissues (highest in the retina and choroid) Not statistically significant changes
**Neuromuscular Blocking Agents**					
**Suxamethonium (Succinylcholine)**	Malthe- Sørenssen et al. [33]	In vivo animal study	IV	Guinea pigs	Low postmortem concentrations in muscle, kidney, and urine Ocular tissues exhibited highest concentrations
**Rocuronium**	N/A	N/A	N/A	N/A	N/A
**Vecuronium**	N/A	N/A	N/A	N/A	N/A
**Opioids**					
**Fentanyl**	Lennborn et al. [24]	Observational	IV	ICU patients	Significant PMR Perimortem concentrations were 5 times higher than observed concentrations in living patients Increase in postmortem compared to perimortem concentrations in 72% of cases
	Brockbals et al. [34]	Observational	IV/transdermal	ICU patients/chronic pain patients	Highest concentrations in liver and kidney L/P ratio was significantly higher than C/P ratio; C/P ratio was also considered high Significant time-dependent increases in peripheral blood Small sample
	Mantinieks et al. [19]	Observational	IV	Patients who died in hospital	Median postmortem/antemortem ratio = 1 Small sample
	Nerdahl et al. [35]	Observational	IV/transdermal	Patients that received fentanyl IV or transdermally	No statistically significant difference for the brain–blood ratios between the two different routes of administration
	McIntyre et al. [36]	Case report	IV	Patient who underwent RSI	C/P: 1.37 Minimal increase in the postmortem concentrations (12%), with a PMI of 12.6 h

^1^ IV, intravenous; BMI, body mass index; C/P, central to peripheral blood; N/A, not available; PMR, postmortem redistribution; L/P, liver to peripheral blood; PMI, postmortem interval.

**Table 3:** Factors that could have mimicked PMR.

Drugs	Factors	Studies	Rationale
Midazolam	Short interval between administration and death	[24,25]	Incomplete distribution
	Sampling from central line	[24]	Central blood is more susceptible to postmortem concentration changes
	Circulatory compromise (shock)	[24,27]	Incomplete distribution
	Critical illness	[24,27]	Incomplete distribution
Fentanyl	Unknown interval between administration and death	[19]	Possible incomplete distribution
	Sampling from central line	[24]	Incomplete distribution
	Circulatory compromise (shock)	[24]	Incomplete distribution
	Critical illness	[24,34]	Incomplete distribution

**Table 4:** Pharmacokinetic properties of RSI drugs and risk for PMR.

Drugs	pKa	logP/ logD	Lipophilic	Hydrophilic	Vd (L/kg)	Protein Binding	Risk for PMR
**Anesthetics**							
Midazolam [25,28,50,51]	6–7	2.73	Highly		1–3	96–97%	Moderate
Thiopental [29,52]	7.55	2.85	Highly		2–3	70–80%	Moderate
Etomidate [30,53]	4.2–4.5	2.8–3	Highly		2–8	75%	High/Uncertain
Propofol [27,31,54]	11	3.8–4.2	Highly		2–12	95–99%	High
Ketamine [32,55]	7.5	2.2–3.1	Highly		3–5	20–50%	High
**Neuromuscular Blocking Agents**							
Suxamethonium (Sucinylcholine) [33,56]	Ionized	−0.74/ −4.15		Highly	0.16–0.5	20%	Low/Uncertain
Rocuronium [56]	7.4	Low/0.54		Highly	0.2–0.27	46%	Low/Uncertain
Vecuronium [56]	8.97	0.9–2/0.75	Poorly	Highly	0.2–0.3	60–80%	Low/Uncertain
**Opioids**							
Fentanyl [19,45,47]	8.4	3.94–4.05	Highly		3–8	80–90%	High

## Data Availability

The data underlying this article will be shared on reasonable request to the corresponding author.

## Abbreviations

The following abbreviations are used in this manuscript:

RSI	Rapid Sequence Induction
RSII	Rapid Sequence Induction and Intubation
PMR	Postmortem Drug Redistribution
ICU	Intensive Care Unit
PMI	Postmortem Interval
CT	Computed Tomography
IV	Intravenous
BMI	Body Mass Index
C/P	Central to Peripheral Blood
L/P	Liver to Peripheral Blood
ADME	Absorption, Distribution, Metabolism, and Excretion
BChE	Butyrylcholinesterase
Vd	Volume of Distribution

## References

[1] Russotto V., Rahmani L.S., Parotto M., Bellani G., Laffey J.G. (2022). Tracheal intubation in the critically ill patient. Eur. J. Anaesthesiol..

[2] Collins J., O’Sullivan E.P. (2022). Rapid sequence induction and intubation. BJA Educ..

[3] Eichelsbacher C., Ilper H., Noppens R., Hinkelbein J., Loop T. (2018). Rapid sequence induction and intubation in patients with risk of aspiration: Recommendations for action for practical management of anesthesia. Anaesthesist.

[4] Sorbello M., Paternò D.S., Zdravkovic I., La Via L. (2025). Pharmacological approach to rapid sequence induction/intubation: A contemporary perspective. Curr. Opin. Anaesthesiol..

[5] Faine B.A., Carroll E., Sharma A., Mohr N. (2024). Rapid sequence intubation, is it time to find an alternative induction agent? A narrative review. J. Pharm. Pract..

[6] Yeung J.K., Zed P.J. (2002). A review of etomidate for rapid sequence intubation in the emergency department. Can. J. Emerg. Med..

[7] Baekgaard J.S., Eskesen T.G., Sillesen M., Rasmussen L.S., Steinmetz J. (2019). Ketamine as a rapid sequence induction agent in the trauma population: A systematic review: A systematic review. Anesth. Analg..

[8] Radkowski P., Szewczyk M., Grażewicz M., Sobolewski K., Onichimowski D. (2025). Use of muscle relaxants in emergency medicine: A review. Med. Sci. Monit..

[9] Amin Aljefri A., Alqurashi A.S., Alshahrani M.F., Alshahrani S.N., Allehaimeed I.S., Alotaibi M.A., Alarifi S.T., Aljohani A.A., Khoj H.A., Samman H.M. (2025). Selection of induction agents for safe and effective rapid sequence intubation. Int. J. Community Med. Public Health.

[10] Bakhsh A., Bakhribah A., Alshehri R., Alghazzawi N., Alsubhi J., Redwan E., Nour Y., Nashar A., Babekir E., Azzam M. (2025). Association between fentanyl use and post-intubation mean arterial pressure during rapid sequence intubation: Prospective observational study. West. J. Emerg. Med..

[11] Gindre S., Ciais J.F., Levraut J., Dellamonica J., Guerin J.P., Grimaud D. (2002). Rapid sequence intubation in emergency: Is there any place for fentanyl?. Ann. Fr. Anesth. Reanim..

[12] De Jong A., Rolle A., Molinari N., Paugam-Burtz C., Constantin J.-M., Lefrant J.-Y., Asehnoune K., Jung B., Futier E., Chanques G. (2018). Cardiac arrest and mortality related to intubation procedure in critically ill adult patients: A multicenter cohort study. Crit. Care Med..

[13] Ali N., Kapadia N.N., Soomar S.M., Raheem A., Habibullah N., Habib Z., Waheed S. (2025). Risk factors of Peri-intubation cardiac arrest in critically ill patients presenting to the Emergency Department of a low-income country: A case-control study. J. Emerg. Med..

[14] Downing J., Yardi I., Ren C., Cardona S., Zahid M., Tang K., Bzhilyanskaya V., Patel P., Pourmand A., Tran Q.K. (2023). Prevalence of peri-intubation major adverse events among critically ill patients: A systematic review and meta analysis. Am. J. Emerg. Med..

[15] Sacco M.A., Gualtieri S., Spiliopoulou C., Tarallo A.P., Verrina M.C., Aquila I. (2025). The role of toxicology investigations in overdose deaths. Cureus.

[16] Musshoff F., Padosch S., Steinborn S., Madea B. (2004). Fatal blood and tissue concentrations of more than 200 drugs. Forensic Sci. Int..

[17] Ferner R.E. (2008). Post-mortem clinical pharmacology. Br. J. Clin. Pharmacol..

[18] Pélissier-Alicot A.-L., Gaulier J.-M., Champsaur P., Marquet P. (2003). Mechanisms underlying postmortem redistribution of drugs: A review. J. Anal. Toxicol..

[19] Mantinieks D., Gerostamoulos D., Glowacki L., Di Rago M., Schumann J., Woodford N.W., Drummer O.H. (2021). Postmortem drug redistribution: A compilation of postmortem/antemortem drug concentration ratios. J. Anal. Toxicol..

[20] Concheiro M., Chesser R., Pardi J., Cooper G. (2018). Postmortem toxicology of new synthetic opioids. Front. Pharmacol..

[21] Pounder D.J., Jones G.R. (1990). Post-mortem drug redistribution--a toxicological nightmare. Forensic Sci. Int..

[22] Irwin M.R., Curay C.M., Choi S., Kiyatkin E.A. (2023). Basic metabolic and vascular effects of ketamine and its interaction with fentanyl. Neuropharmacology.

[23] Wiczling P., Bieda K., Przybyłowski K., Hartmann-Sobczyńska R., Borsuk A., Matysiak J., Kokot Z.J., Sobczyński P., Grześkowiak E., Bienert A. (2016). Pharmacokinetics and pharmacodynamics of propofol and fentanyl in patients undergoing abdominal aortic surgery—A study of pharmacodynamic drug-drug interactions: PK/PD OF PROPOFOL AND FENTANYL DURING ABDOMINAL AORTIC SURGERY. Biopharm. Drug Dispos..

[24] Lennborn U., Johansson A., Lindgren E., Nielsen E.I., Sandler H., Kronstrand R., Ahlner J., Kugelberg F.C., Rubertsson S. (2025). Comparison of pre-mortem and post-mortem blood concentrations of analgesic and sedative drugs in intensive care patients. Forensic Sci. Int..

[25] Oertel R., Pietsch J., Arenz N., Zeitz S.G., Goltz L., Kirch W. (2011). Distribution of metoprolol, tramadol, and midazolam in human autopsy material. J. Chromatogr. A.

[26] de Groot A.D.E., Borra L.C.P., van der Hulst R., Etsouli O., Kloos D.-P., Rijken D.J., Elsinga P.H., Boersma H.H., Bosman I.J., Touw D.J. (2023). Postmortem redistribution of amphetamines and benzodiazepines in humans: Important variables that might be influencing the central blood / peripheral blood ratio. Forensic Sci. Int..

[27] Tominaga M., Michiue T., Ishikawa T., Kawamoto O., Oritani S., Ikeda K., Ogawa M., Maeda H. (2013). Postmortem analyses of drugs in pericardial fluid and bone marrow aspirate. J. Anal. Toxicol..

[28] Gerostamoulos D., Beyer J., Staikos V., Tayler P., Woodford N., Drummer O.H. (2012). The effect of the postmortem interval on the redistribution of drugs: A comparison of mortuary admission and autopsy blood specimens. Forensic Sci. Med. Pathol..

[29] Yasudal T., Yamaba T., Sawazaki K., Masuyama F., Nadano D., Takeshita H., Kishi K. (1993). Postmortem concentrations of thiopental in tissues: A sudden death case. Int. J. Leg. Med..

[30] Molina D.K., Hargrove V.M., Rodriguez R.G. (2008). Distribution of etomidate in a fatal intoxication. J. Anal. Toxicol..

[31] George A.A., Hargrove V.M., Molina D.K. (2016). Postmortem propofol levels: A case of residual detection long after administration. Am. J. Forensic Med. Pathol..

[32] Arora B., Lalwani S., Saxena R., Ghose S., Velpandian T. (2020). Postmortem redistribution of ketamine in ocular matrices: A study of forensic relevance. Leg. Med..

[33] Malthe-Sørenssen D., Odden E., Blanch J., Bugge A., Mørland J. (1986). Determination of succinyldicholine in different tissue samples from guinea pigs after injection of a single intravenous dose. Forensic Sci. Int..

[34] Brockbals L., Staeheli S.N., Gascho D., Ebert L.C., Kraemer T., Steuer A.E. (2018). Time-dependent postmortem redistribution of opioids in blood and alternative matrices. J. Anal. Toxicol..

[35] Nedahl M., Johansen S.S., Linnet K. (2021). Postmortem brain-blood ratios of codeine, fentanyl, oxycodone and tramadol. J. Anal. Toxicol..

[36] McIntyre I.M., Gary R.D., Estrada J., Nelson C.L. (2014). Antemortem and postmortem fentanyl concentrations: A case report. Int. J. Leg. Med..

[37] Olson K.N., Luckenbill K., Thompson J., Middleton O., Geiselhart R., Mills K.M., Kloss J., Apple F.S. (2010). Postmortem redistribution of fentanyl in blood. Am. J. Clin. Pathol..

[38] Luckenbill K., Thompson J., Middleton O., Kloss J., Apple F. (2008). Fentanyl postmortem redistribution: Preliminary findings regarding the relationship among femoral blood and liver and heart tissue concentrations. J. Anal. Toxicol..

[39] Palamalai V., Olson K.N., Kloss J., Middleton O., Mills K., Strobl A.Q., Thomas L.C., Apple F.S. (2013). Superiority of postmortem liver fentanyl concentrations over peripheral blood influenced by postmortem interval for determination of fentanyl toxicity. Clin. Biochem..

[40] Rodney G., Raju P., Brull S.J. (2024). Neuromuscular block management: Evidence-based principles and practice. BJA Educ..

[41] Grissinger M. (2019). Paralyzed by Mistakes—Reassess the Safety of Neuromuscular Blockers in Your Facility. Pharm. Ther..

[42] Perše M. (2024). Animal Models of Human Pathology: Revision, relevance and refinements. Biomedicines.

[43] Leenaars C.H.C., Kouwenaar C., Stafleu F.R., Bleich A., Ritskes-Hoitinga M., De Vries R.B.M., Meijboom F.L.B. (2019). Animal to human translation: A systematic scoping review of reported concordance rates. J. Transl. Med..

[44] Zagalioti S.-C., Gkarmiri S., Karagiannidis E., Stachteas P., Zgouridou A., Zagaliotis P., Kotzampassi K., Grosomanidis V., Raikos N., Aggou M. (2025). Does the injection site matter during CPR? A systematic review and meta-analysis of drug pharmacokinetics and pharmacodynamics. J. Clin. Med..

[45] McIntyre I.M. (2012). Postmortem Fentanyl Concentrations: A Review. J. Forensics Res..

[46] Kuip E.J.M., Zandvliet M.L., Koolen S.L.W., Mathijssen R.H.J., van der Rijt C.C.D. (2017). A review of factors explaining variability in fentanyl pharmacokinetics; focus on implications for cancer patients. Br. J. Clin. Pharmacol..

[47] Bird H.E., Huhn A.S., Dunn K.E. (2023). Fentanyl absorption, distribution, metabolism, and excretion: Narrative review and Clinical significance related to illicitly manufactured fentanyl. J. Addict. Med..

[48] Stephenson L., Van Den Heuvel C., Scott T., Byard R.W. (2024). Difficulties associated with the interpretation of postmortem toxicology. J. Anal. Toxicol..

[49] Peter J.-U., Dieudonné P., Zolk O. (2024). Pharmacokinetics, pharmacodynamics, and side effects of midazolam: A review and case example. Pharmaceuticals.

[50] Greenblatt D.J., Abernethy D.R., Locniskar A., Harmatz J.S., Limjuco R.A., Shader R.I. (1984). Effect of age, gender, and obesity on midazolam kinetics. Anesthesiology.

[51] National Center for Biotechnology Information PubChem Compound Summary for CID 4192, Midazolam. https://pubchem.ncbi.nlm.nih.gov/compound/Midazolam.

[52] National Center for Biotechnology Information PubChem Compound Summary for CID 3000715, Thiopental. https://pubchem.ncbi.nlm.nih.gov/compound/Thiopental.

[53] Levron J.C., Assoune P. (1990). Pharmacocinétique de l’étomidate. Ann. Fr. Anesth. Reanim..

[54] Sahinovic M.M., Struys M.M.R.F., Absalom A.R. (2018). Clinical Pharmacokinetics and Pharmacodynamics of Propofol. Clin. Pharmacokinet..

[55] Dinis-Oliveira R.J. (2017). Metabolism and metabolomics of ketamine: A toxicological approach. Forensic Sci. Res..

[56] Roy J.J., Varin F. (2004). Physicochemical properties of neuromuscular blocking agents and their impact on the pharmacokinetic-pharmacodynamic relationship. Br. J. Anaesth..

[57] McIntyre I.M. (2014). Liver and peripheral blood concentration ratio (L/P) as a marker of postmortem drug redistribution: A literature review. Forensic Sci. Med. Pathol..

[58] Cheng T., Curley M., Barmettler A. (2024). Pseudocholinesterase deficiency in ophthalmology: A systematic review. Orbit.

[59] Akan M., Çakırgöz M., Demirel İ., Saraç Ö., Kar A.A., Alaygut E., Demirel O., Yeniay H., Tünay A. (2025). Thiopental Versus Propofol in Combination with Remifentanil for Successful Classic Laryngeal Mask Airway Insertion: A Prospective, Randomised, Double-Blind Trial. Pharmaceuticals.

